# Gradient-based feature-attribution explainability methods for spiking neural networks

**DOI:** 10.3389/fnins.2023.1153999

**Published:** 2023-09-27

**Authors:** Ammar Bitar, Rafael Rosales, Michael Paulitsch

**Affiliations:** ^1^Intel Labs, Munich, Germany; ^2^Department of Knowledge Engineering, Maastricht University, Maastricht, Netherlands

**Keywords:** spiking neural networks, explainable artificial intelligence, XAI, brain-inspired computation, neuromorphic, event-based encoding, MNIST, CIFAR

## Abstract

**Introduction:**

Spiking neural networks (SNNs) are a model of computation that mimics the behavior of biological neurons. SNNs process event data (spikes) and operate more sparsely than artificial neural networks (ANNs), resulting in ultra-low latency and small power consumption. This paper aims to adapt and evaluate gradient-based explainability methods for SNNs, which were originally developed for conventional ANNs.

**Methods:**

The adapted methods aim to create input feature attribution maps for SNNs trained through backpropagation that process either event-based spiking data or real-valued data. The methods address the limitations of existing work on explainability methods for SNNs, such as poor scalability, limited to convolutional layers, requiring the training of another model, and providing maps of activation values instead of true attribution scores. The adapted methods are evaluated on classification tasks for both real-valued and spiking data, and the accuracy of the proposed methods is confirmed through perturbation experiments at the pixel and spike levels.

**Results and discussion:**

The results reveal that gradient-based SNN attribution methods successfully identify highly contributing pixels and spikes with significantly less computation time than model-agnostic methods. Additionally, we observe that the chosen coding technique has a noticeable effect on the input features that will be most significant. These findings demonstrate the potential of gradient-based explainability methods for SNNs in improving our understanding of how these networks process information and contribute to the development of more efficient and accurate SNNs.

## 1. Introduction

Conventional neural networks, also known as artificial neural networks, although proven to be useful for numerous tasks, have latency, energy and storage requirements that may not be adequate for many real-time tasks in energy-constrained environments. The field of neuromorphic computing, inspired by the efficiency of biological neural networks, attempts to replicate this efficiency through brain-inspired models of computation such as SNNs (Maass, 1997).

The base substrate of communication in biological neurons are parallel, asynchronous and discrete action-potentials or more commonly: *spikes* (Kress and Mennerick, 2009). Spikes can be regarded as an “all-or-none” binary format, where the information is transmitted in the timings between the spikes rather than in the spike magnitude as in real-valued data. SNNs process these spikes by integrating them over time. The dynamics of an SNN are governed by the spiking neuron model, such as the *Spike Response Model* (SRM) (Gerstner, 1995; Gerstner et al., 2014) and the *Leaky Integrate-and-Fire* (LIF) model Gerstner et al. (2014). These neuron models use a thresholding function – the spiking function – as an analog to the activation function in ANNs. Spiking neurons integrate incoming spikes over time, and only fire spikes themselves when their inner potential reaches a predefined threshold. Thus, contrary to ANNs, whose input traverses the whole network, SNNs deeper neurons only activate when previous neurons integrated enough spikes to propagate. This results in less neuron activations for SNNs than ANNs. Optimally, SNNs are paired with event-based sensors, which naturally generate sparse spiking data at thousands of frames per second (FPS), such as event-based cameras or spiking cochlear sensors (Vanarse et al., 2016; Gallego et al., 2022). Pragmatically, conventional (real-valued) data, such as 2D images, can be encoded into the spiking domain allowing its processing using SNNs as well. To enable an efficient processing of SNNs, several neuromorphic chips have been developed (Akopyan et al., 2015; Davies et al., 2018, 2021; Haessig et al., 2019; Pehle et al., 2022) that provide a native hardware computing platform.

The focus on SNNs in recent research has been on addressing challenges related to energy consumption, performance, and interpretability. Notably, two recent studies have proposed novel frameworks for improving SNN performance in specific tasks. The first study introduces a spike-based framework, MeMEE, which utilizes minimum error entropy to enhance online meta-learning performance in SNNs (Yang et al., 2022a). This approach emphasizes the integration of advanced information theoretic learning methods into spike-based learning algorithms. Similarly, the second study presents HESFOL, a heterogeneous ensemble-based spike-driven few-shot online learning framework that leverages entropy theory to enhance few-shot learning performance in SNNs (Yang et al., 2022b). These studies collectively contribute to the ongoing efforts to optimize SNNs for various learning tasks.

While there are some methods available to explain the decisions made by SNNs, there are still significant gaps in our understanding of these networks. One of the key challenges is the lack of effective *Explainability methods* for SNNs. In Section 1.2.1, we discuss some approaches that have been proposed to explain SNN inference results, such as attribution maps. Attribution maps provide a visualization of the input features that are most important to the SNN's inference decision. However, none of the existing approaches for SNNs can provide true attributions, which are a mechanism to accurately and efficiently compute the contribution of each input feature to the network's output. Moreover, existing methods are not applicable to all types of layers, and they cannot be used *post hoc* without retraining or supplementing the model. Therefore, there is a need for more robust Explainability methods for SNNs that can address these challenges and provide true attributions.

**Figure 1:**
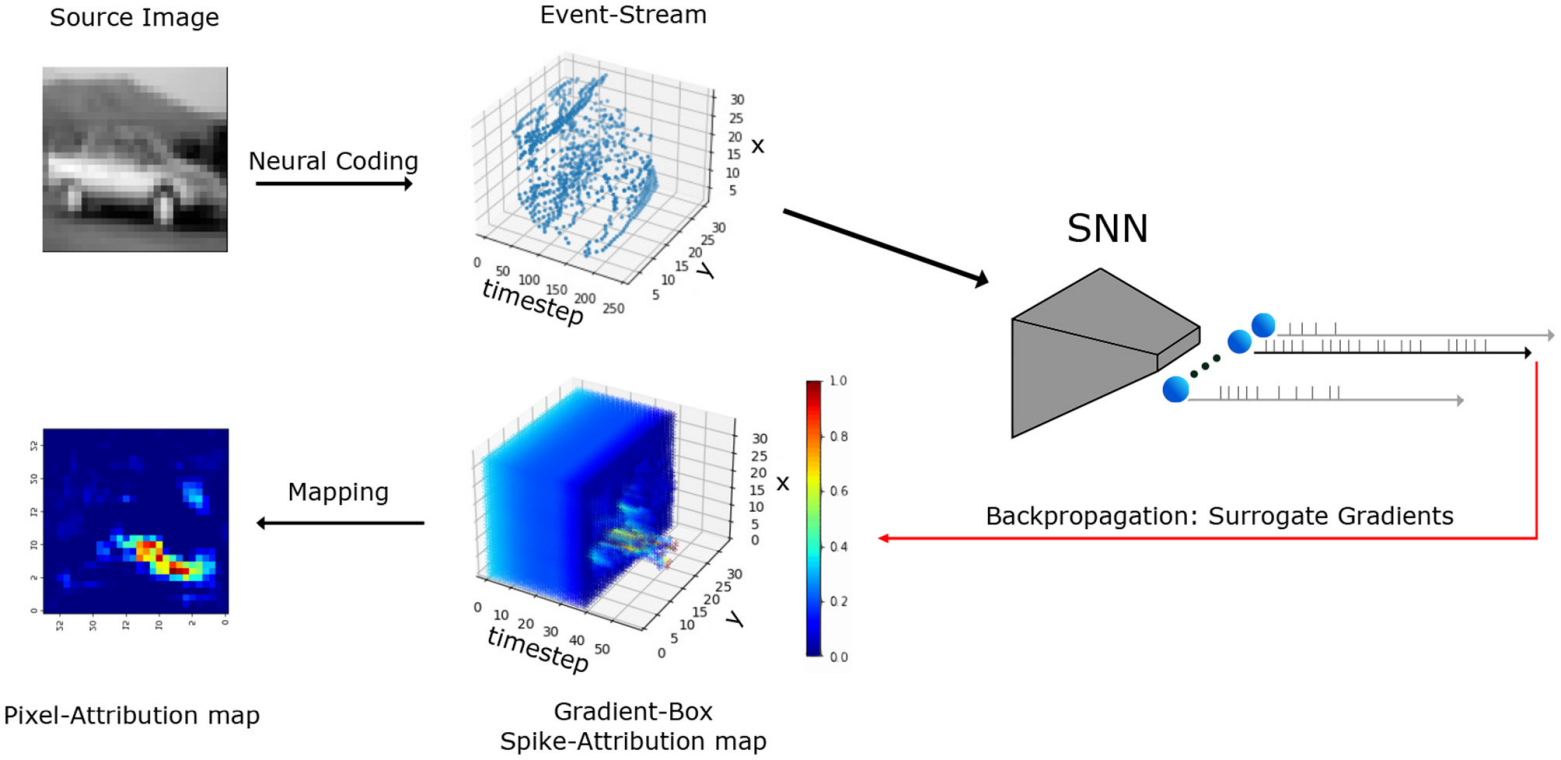
Illustration of general computational flow for explainability attribution methods for SNNs. Spike- and pixel-level attribution maps (3D and 2D, respectively) are generated for an inference result of a two dimensional image input. The methodology is based on surrogate gradients to identify the most important input spikes to the SNN. Neural coding strategies are used to transform an image to a spiking representation and mapping strategies are finally applied to reduce the 3D spike attributions to the original 2D image. Iterative inference runs on interpolated inputs at pixel or spike-level performed by advanced attribution methods are not shown here for clarity.

The work at hand explores, for the first time, gradient-based attribution methods for SNNs trained with the backpropagation approach. Figure 1 illustrates an end-to-end flow for SNN attribution map generation, starting from a 2D image and producing both 3D spike-domain and 2D pixel attribution maps. We perform our experiments on image classification on conventional 2D data encoded into the spiking domain through neural codings as well as on event-based data obtained from an event-based camera. To note, our methods are not specific to image classification, and can be used to explain the input feature importance for any model trained with an SNN backpropagation approach.

The major contributions of this work are the following:

1. We evaluate the efficacy of the vanilla Saliency method (Simonyan et al., 2014) on SNNs for spiking event-data to obtain input spike-attribution maps. This method will be referred to as SNN-Grad3D. Saliency in ANNs computes the gradient of the output neuron with the highest score with respect to input changes. Our proposed method adapts this procedure for SNNs by computing a *surrogate* gradient of the output, this time consisting in the neuron with the *highest number* of binary spikes w.r.t to a change to an *input spike*.
2. We propose SNN-IG3D: a novel adaptation of the Integrated Gradients (IG) method (Sundararajan et al., 2017) for spiking event-data, to obtain input spike-attribution maps. IG in ANNs computes the vanilla saliency across many interpolations between the image and a baseline to afterwards integrate them. Our proposed method adapts the idea of a path of integration to the time dimension, where the sampling of input spikes according to a given heuristic is performed.
3. We propose an efficient generation of Saliency and IG attribution maps for SNNs with real-valued inputs (e.g., pixel-level for 2D input images) through (a) spike encoding, (b) spike-attribution map generation, and (c) mapping from spike-attribution map to an attribution map of the input. These methods are referred to as SNN-Grad2D and SNN-IG2D. Note that although the end result is the same as a typical ANN attribution map, i.e., a 2D image, an SNN operates with one discrete dimension more and thus traditional ANN methods cannot be directly used in SNNs.
4. We qualitatively assess our methods through visual inspection, giving an insight on how SNNs classify with different neural encodings and demonstrate through quantitative perturbation analysis that the resulting attribution maps for both spiking event-based data and real-valued 2D images are quantitatively superior to the alternative *post hoc* existing approaches for SNNs.

### 1.1. Background and related work: neuromorphic computing

Spiking neural networks are biologically inspired neural networks, which possess similar topology as artificial neural network but differing in terms of base neuron units. Information is transmitted across neurons through the synapses connecting pre- and post-synaptic neurons. Incoming spikes are integrated into the neurons' *membrane potential*. As the membrane potential reaches a critical *threshold*, the neuron fires a spike to the next connected neurons and the potential is reset. A positive membrane potential is continuously reduced, or *leaked*, so the timing of the incoming spikes is critical. SNNs aim to reproduce these properties of biological neurons using differential equations for the neuron models and consuming and producing spiking data rather than floating point numbers as in conventional ANNs. As the spiking neurons only propagate spikes after integrating multiple incoming spikes, data passed through the SNN is sparse, and thus a great number of neurons remain silent during a forward pass. These are the main reasons for the sparsity and energy-efficiency of SNNs. Please refer to the Appendix 3 for a concise formal description of the neuron model. There are nowadays three different approaches to create a trained SNN:

#### 1.1.1. Spike-timing dependant plasticity

Unsupervised learning based on timing causality of pre- and post-synaptic spikes: if an input spike preceded an output spike it probably had a causal relationship. STDP has been successfully used to train a variety of shallow SNNs (Hu et al., 2014; Hao et al., 2020). However, scaling up STDP training to deeper networks remains cumbersome, requiring training each layer individually (Kheradpisheh et al., 2018; Lee et al., 2018) resulting in a large training time.

#### 1.1.2. ANN-to-SNN conversion

Takes a pretrained ANN model and converts all neurons to spiking neurons and then converts the ANN weights to SNN weights keeping the activation patterns of the neurons similar between ANN ReLU neurons and spiking neurons. It has been used to generate trained SNNs for multiple tasks and models with great success (Sengupta et al., 2019). However, Lee et al. (2020) has shown that spike efficiency, is vastly reduced for SNNs trained with this method, as converted SNNs require a large amount of timesteps to reach sufficient inference power, compared to other methods.

#### 1.1.3. Backpropagation using a surrogate function

As the spiking function is not inherently differentiable (Bellec et al., 2018; Zenke and Ganguli, 2018; Zenke and Vogels, 2021), surrogate functions have been used to approximate its derivative. Surrogate functions for backpropagation have been extensively used to train shallow and deep-SNNs on many tasks, such as classification tasks on images (MNIST, CIFAR-10,...) and on neuromorphic event-based data (DVSGesture, N-MNIST, CIFAR10-DVS) (Shrestha and Orchard, 2018; Neftci et al., 2019; Li et al., 2021). While existence of a backpropagation mechanism in the brain remains contested; backpropagation for SNN training gives us access to well-established state-of-the art optimization procedures (Kingma and Ba, 2015) for ANN which can then be directly used to train the SNN, enabling training of deep-SNNs in an efficient manner. We take advantage of this training approach in our work, to research explainability methods for deep-SNNs.

### 1.2. Background and related work: explainable AI

Within the vast literature on explainable artificial intelligence (XAI) for neural networks, we restrict the discussion solely to input attribution and layer activation methods for image data, and present the existing XAI methods for spiking neural networks. XAI methods can be divided into two categories; (i) model-agnostic and (ii) model-specific methods.

Model-agnostic methods, such as LIME (Ribeiro et al., 2016) and RISE (Petsiuk et al., 2018), treat the model as a black-box and explain the models' decision by perturbations, such as occlusion, at the input. These methods can be readily applied to SNNs as they are independent of their inner-workings. However, model-agnostic methods are very computationally intensive, and thus slow and typically configured to provide coarse-grained results due to their poor scalability as the perturbation experiments are combinatorial.

On the other hand, model-specific methods make use of the knowledge we have of the model to generate explanations. Known model-specific approaches for ANNs are deconvolution models (Zeiler and Fergus, 2014) created during training (Zhou et al., 2015) or (*post hoc*) gradient-based approaches, such as Saliency Maps (Simonyan et al., 2014), Integrated Gradients (Sundararajan et al., 2017), Grad-CAM (Selvaraju et al., 2017), and other extensions, (e.g., Chattopadhay et al., 2018; Srinivas and Fleuret, 2019).

#### 1.2.1. Explainable neuromorphic computing

For spiking neural networks, however, research into their explainability and interpretability is still rather sparse. Some initial studies have been done initiating and coining research into explainable neuromorphic computing. Kirkland et al. (2020) take inspiration from Zeiler and Fergus (2014) to create a spike segmentation algorithm by extending their spiking convolutional neural network with extra deconvolution layers to reconstruct the segmentation map. They provide experimental results using STDP for training on both spiking and spike-encoded datasets. The main disadvantage of this approach is the necessity to train the additional deconvolutional layers. In Kim and Panda (2021), two explainability methods for SNNs are proposed: SNN-crafted Grad-CAM and Spike Activation Map (SAM). Both methods produce *activation maps* for SNNs, i.e., which input regions triggered a layer the most. The former uses backprop up to the convolutional layer of interest, while the latter only forward propagates the effect of a spike to successive layers. Afterwards, the activation maps at particular convolutional layers can then be scaled to the input image size for superposition. These two approaches to create visual 2D feature maps are however limited to convolutional layers. In this work, we evaluate through input perturbation experiments the efficacy of SAM as a tool to obtain input attribution maps as Kim and Panda (2021) only validated its results against the output of Grad-CAM as ground truth.

#### 1.2.2. Metrics for attribution maps

Most explainability methods have been evaluated through subjective visual inspection susceptible to human interpretation bias. In recent years, less biased quantitative methods for evaluation of explainability methods based on input perturbation have begun to appear (Ancona et al., 2017; Montavon et al., 2017; Samek et al., 2017; Petsiuk et al., 2018). Generally, these methods work by iterativeley perturbing the most or least salient pixels and then re-evaluating the models performance on the perturbed input. Other methods, such as RemOve And Retrain (ROAR) (Hooker et al., 2019) retrain the model after each iteration of salient-input perturbation, and then evaluate the resulting models' performance. In this paper, we evaluate using deletion and insertion perturbation experiments without retraining, as, besides the huge computation savings, the accuracy degradation of retrained models on perturbed inputs indicates the *potential* of input features to be informative as ANN models are able to perform a good job by re-training with very few inputs (Hooker et al., 2019), whereas the accuracy degradation of the same (not retrained) model on perturbed inputs informs us about input feature importance of the model under test.

## 2. Materials and methods

This section presents the theoretical motivation, data, neural codings, SNN-crafted gradient-based XAI methods, mapping of spiking attribution map to real-valued input attribution map, and experimental setup used in our study on neuromorphic computing. The goal of this study is to investigate the use of spiking neural networks for image classification tasks and to develop gradient-based explainable AI methods that can be applied to SNNs. In particular, we focus on using SNNs to classify images from the Cifar 10 and DVS128 Gesture Dataset, and developing methods to understand the decisions made by SNNs through the use of attribution maps.

In Section 2.3 and onwards, we present our approach to obtain spike- and pixel-level attribution maps for 2D input images. The methodology comprises a three-phase system (refer to Figure 1), which involves: (1) encoding 2D input images into a 3D event stream (discussed in Section 2.3), (2) computing the 3D input spike attributions of the SNN's output (detailed in Section 2.4), and (3) mapping the 3D spike attribution to a 2D pixel-attribution map that corresponds to the original input image (elaborated in Section 2.4.3). We also present our methods for event input data, which does not require the initial encoding and final mapping parts. Our first method, SNN-Grad3D, employs gradients to compute 3D attributions across time, and to compute pixel-level attributions, we propose SNN-Grad2D. For our second group of methods, SNN-IG (2D and 3D), based on Integrated Gradients, we compute the 3D attributions using a time-based sampling heuristic applied on the input data. In Section 2.6.1, we present the experimental setup, including SNN architectures, the employed training methodology and the evaluation process for the resulting explanations.

### 2.1. Theoretical motivation

Our hypothesis is that gradient-based methods will enable to obtain better attribution maps than activation-based methods for SNNs, as the gradient encapsulates the knowledge of the changes in the input that are needed to see a change in the output. Activation maps do not capture this relationship necessarily. ANN's Class Activation Maps (CAM) had to be trained to learn that relationship, and GradCAM introduced gradient back propagation to estimate an attribution map from pure activations. Furthermore, a better model-specific attribution method will also allow to avoid the huge cost of model-agnostic computational combinatorial explosion.

### 2.2. Data

The experiments in this study were conducted using two datasets: Cifar 10 and DVS128 Gesture Dataset.

Cifar-10 (Krizhevsky, 2009) is a widely used dataset for image classification tasks, consisting of 60,000 32 × 32 color images in 10 classes, with 6,000 images per class. There are 50,000 training images and 10,000 test images. It is important to note that the RGB color images were first grayscaled and then scaled to a range of 0–255 before being used in the experiments.

DVS128 Gesture Dataset (Amir et al., 2017)is a dataset for gesture recognition tasks, consisting of events generated by a Dynamic Vision Sensor (DVS) with a 128 × 128 resolution. The dataset contains 10 different gestures, each performed by 5 different subjects, for a total of 50 gestures per class.

### 2.3. Neural codings for real-valued input data (2D images)

The input of SNNs requires to be in the spiking domain. In this work, we explored various neural codings, with varying degrees of temporal and spatial complexity. These different encodings distribute the input information differently throughout the spiking domain, e.g., a pixel value could be conveyed as a single spike, or as a rate of spikes; potentially influencing the computation of input attributions.

#### 2.3.1. Time To first spike

TTFS (Park et al., 2020) is a form of latency coding which encodes each input pixel-value into a corresponding spike occurring at a timestep proportional to the pixel value. This encoding results in the sparsest input-encoding and lowest temporal complexity, as any pixel will have a singular spike. In this work, the grayscale resolution is maintained and thus TTFS spike-timings fall in a window between 0 and 255 timesteps. The description and equations of TTFS and further neural codings, not discussed here due to space constraints, can be found in the Appendix 4.

#### 2.3.2. Comparison of encodings based on their robustness to hardware noise

Additionally, we conducted a comparative analysis of different neural encodings in terms of their robustness to noise encountered in realistic scenarios, including noise from neuromorphic sensor recordings and noise within neuromorphic chips (Park et al., 2021; Nagarajan et al., 2022).

The subsequent paragraphs describe the experiments conducted to assess the robustness of various neural encodings to noise. Drawing inspiration from Park et al. (2021), we designed experiments where input spikes are perturbed using two noise models: spike deletion and jitter, the latter of which introduces temporal fluctuations to spike timing. Spike deletion can emulate scenarios where a sensor fails to generate spikes or an input neuron fails to fire, while jitter can be caused by various factors such as spike transmission malfunctions or altered voltage thresholds of spike functions.

##### 2.3.2.1. Effect of input noise

We replicated the experiment from Park et al. (2021) by varying the intensity of jitter from 0 to 4 time steps, allowing spikes to occur earlier or later. Spike deletion noise was modeled with a probability of spike deletion for each spike. We performed a sweep of deletion probabilities from 0% to 90%.

**Figure 2:**
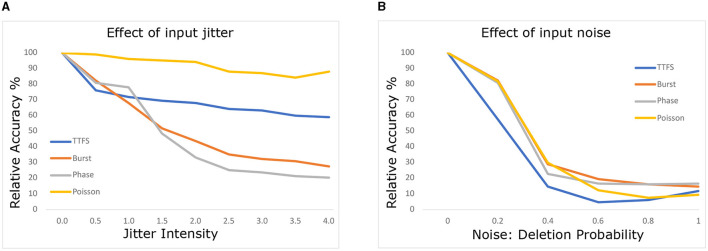
Effects of **(A)** jitter and **(B)** deletion noise at the input level on model accuracy.

Figure 2 illustrates the results for (a) jitter and (b) deletion noise. Following the application of jitter or deletion noise, we measured the relative accuracy of the model. Relative accuracy is defined as the network's accuracy compared to its accuracy on the original non-perturbed data. The analysis focused on a subset of data correctly classified by all four neural encodings to maintain parity in input samples, accounting for variations in network accuracy. Although network accuracy varied across different encodings for CIFAR10, we used this approach to ensure fairness, acknowledging that bias might be introduced due to selective sample picking.

Consistent with Park et al. (2021), we observed strong robustness of Poisson coding against jitter noise. We attribute this to the inherent stochastic nature of Poisson coding, which manifests during training. Interestingly, our experiments indicate greater robustness of TTFS against jitter noise than previously reported. Even at maximum jitter intensity, the relative accuracy of TTFS-SNN only drops to 60% accuracy, contrasting with the 10% reported in the original study. We speculate that this difference could arise from methodological variations, including the training approach and the reduced number of time steps employed in the original TTFS study.

Regarding deletion noise, our findings align with Park et al. (2021), except for TTFS, which displayed higher vulnerability to deletion noise in our experiments compared to its reported robustness. We hypothesize that the reduced number of time steps used in the original TTFS study could explain this discrepancy.

##### 2.3.2.2. Effect of network noise

**Figure 3:**
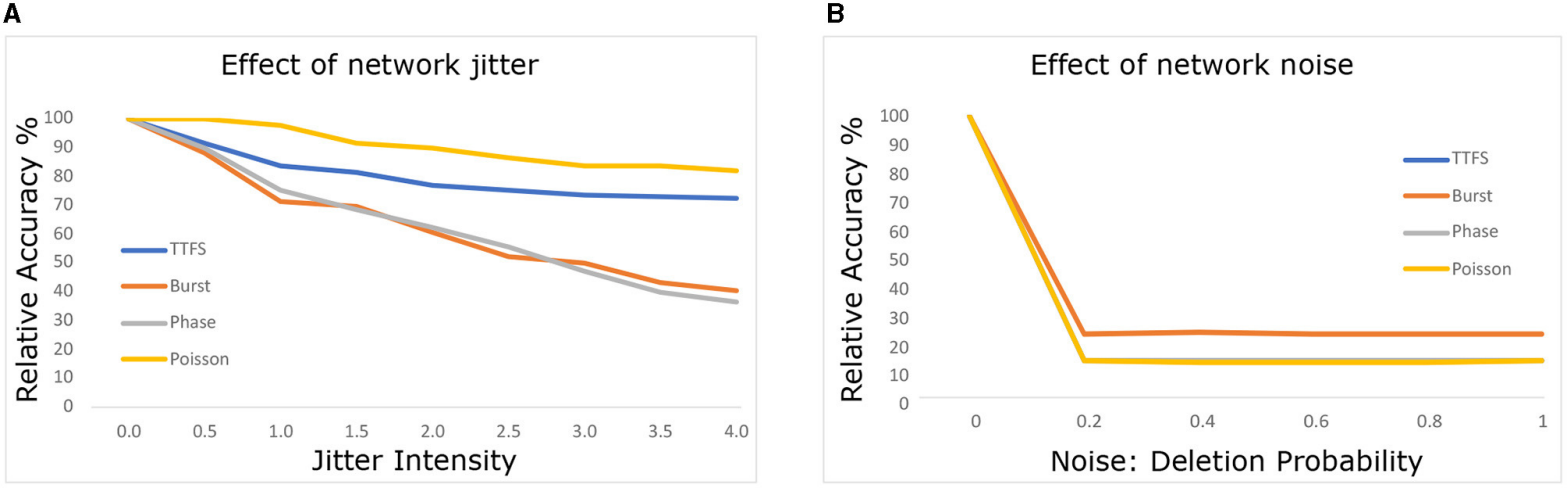
Effects of **(A)** jitter and **(B)** deletion noise at the network level on model accuracy.

In Figure 3, we explore the SNNs' robustness to noise at the network level, excluding input noise. We introduce similar noise models, but this time perturbing layers and neurons throughout the network instead of limiting the perturbation to input data. Neuromorphic hardware, as discussed in Nagarajan et al. (2022), can experience such noise due to external attacks or electrical interference.

Consistent trends emerged, with Poisson and TTFS encodings maintaining robustness against jitter noise throughout the network. Additionally, phase and burst coding exhibited improved jitter robustness within the network compared to the input. We hypothesize that network jitter is less disruptive due to the dense spike traffic within the network, which stabilizes membrane potential levels.

Conversely, deleting just 20% of spikes within the network led to significant performance degradation. Spike deletion within the network appears more effective at disrupting network operation. This outcome aligns with expectations, as spike deletion encourages leakage of membrane potential, leading to reduced spike frequency and neuron activation within the network. In contrast, jitter affects spike timing without necessarily promoting leakage, thereby exerting a milder impact.

### 2.4. SNN-crafted gradient-based XAI methods

Based on established gradient-based pixel-attribution methods for ANNs, we propose two classes of SNN-Crafted counterparts, namely SNN-Grad (*Gradients*) and SNN-IG (*Integrated Gradients*) to obtain spike-level and pixel-level attribution maps. For all methods, to compute the gradients through the post inference backward pass, we use a surrogate-gradient approach, as the spiking function is non-differentiable and thus an approximation is necessary.

#### 2.4.1. SNN-Gradients (SNN-Grad)

Saliency maps, first introduced by Simonyan et al. (2014), compute the gradient of class probability with respect to the input values, i.e., it allows to determine how much a specific output for one class changes if an input feature (pixel) is slightly changed. In SNNs trained through backpropagation, a classification decision is typically obtained by selecting the output class with the highest amount of spikes in a time window as all other classes are expected to produce a pre-defined reduced amount of spike activity too. This is the expected behavior induced during training for convergence reasons.

##### 2.4.1.1. SNN-Grad3D for event-based data

We define our SNN-Grad3D similarly to the original Saliency Map (Simonyan et al., 2014). As the output of the SNN is not a logit or a class score, but rather a spike train, we define our class score (*S*_*C*_) as the sum of spikes per output neuron. Indeed, the classification decision of an SNNs output is usually decided by the most spiking output neuron. Thus output neuron with the highest number of spikes is naturally the winning class. We define our SNN-Grad3D map to represent the gradients of the class score (*S*_*C*_) with respect to the input. Using the surrogate-gradient, for an image of size *height*×*width* encoded in *t* timesteps, we obtain a spike attribution map of size *height*×*width*×*t* indicating how much would the class score *S*_*C*_ change if a spike could be added or removed at any point in this 3D space.

##### 2.4.1.2. SNN-Grad2D for real-valued data

To obtain an attribution map for 2D images SNN-Grad3D is simply combined with a mapping strategy (Section 2.4.3), to reduce the temporal dimension.

#### 2.4.2. SNN-Integrated Gradients (SNN-IG)

The second set of proposed gradient-based attribution methods, are an adaptation of the Integrated Gradients (Sundararajan et al., 2017) approach. The original Integrated Gradients aims to provide better attribution maps than the simple Saliency approach by solving the locality problem of a gradient only reflecting changes of the output due to *small* variations of the input. It achieves this by first creating a monotonous path from a *baseline* input to the original input in *n_steps*. IG is then computed by integrating gradients along this path.

##### 2.4.2.1. Choice of baseline

Usually, pixels values are set to black in conventional 2D images (Sundararajan et al., 2017; Petsiuk et al., 2018). However, replacing of pixels with black ones creates artifacts which may start being detected by the model (Srinivas and Fleuret, 2019). In spiking data, e.g., with TTFS encoding, each pixel-value can be assigned a specific spike-timing. The equivalent of a black pixel is in this case a spike at the earliest time. On the other hand, event-based cameras generate spikes only when movement is detected, thus deleting spikes directly translates to missing information. Therefore, for our spike-level experiments on our 2D system, we used an encoded black baseline, and for spiking data, we used “no spike” as the baseline.

As event stream inputs are binary timeseries, intermediary steps between “off” and “on” spikes do not exist, thus a path cannot be created by *reducing spike values* (contrary to pixels which can be, e.g., dimmed). However, recall that the information is encoded across spikes in the timing *between* the spikes. For this work, we adapt IG into two SNN-Crafted IG versions depending on the original input: 2D image (SNN-IG2D) and event-based sensor (SNN-IG3D).

##### 2.4.2.2. SNN-IG2D for real-valued data

Leveraging our knowledge of the original 2D input data, similarly to Integrated Gradients (Sundararajan et al., 2017) we create a path from a fully black baseline image to the source (2D) image. We then encode every single image along the path into event streams, compute the individual SNN-Gradients with respect to the input and integrate them along that path.

##### 2.4.2.3. SNN-IG3D for event-based data

Contrary to the 2D image procedure, event-based data exists purely in the binary spiking domain and does not require an encoding. Thus, as the individual spikes cannot be further divided to create a straight path from a baseline, we adapt our approach to create a path along the time-dimension. Inspired by the temporal and sequential properties of the event-based data (similar to video data), we create a path from an empty 3D event stream, to the original input event-data sequence by sampling timesteps from the original event-data input with iteratively increasing sampling-rate along the path, up to the complete original event stream over *i* steps. Doing this, we end-up with *i* event streams creating a path from a baseline to the original data. Details and visualization of the two sampling-rate approaches explored to create path of event-based data can be found in Appendix 10. Similarly, we then compute the individual SNN-Gradients with respect to these new inputs, and integrate them along the path.

#### 2.4.3. Mapping of spiking attribution map to real-valued input attribution map

To close the explanation loop for real-value input data, a mapping from the spike-level attribution-box to the original input space is necessary (see Figure 1). For 2D image data, this corresponds to a mapping from a 3D attribution-box to a 2D attribution map. In this work, we explored various basic mappings, such as max, sum, and avg, to aggregate the gradients across the three dimensional space (height, width, and time) to the two dimensional space (height, width). For the following presented experiments, we chose the best performing mapping on SNN-Grad2D and SNN-IG2D, which was the summation across time for both. Further mappings and their equations can also be found in the Appendix 9.

### 2.5. Inverted SAM

In this subsection, we introduce Inverted SAM (ISAM), a modified version of the SAM method used in the perturbation experiments for interpreting the encoded event stream. ISAM aims to enhance the attribution mapping performance specifically in the context of spiking neural networks.

ISAM is derived by taking the negative of the SAM scores obtained from the convolutional layers of the SNN. This inversion of scores provides an alternative perspective by highlighting regions that are deemed less important or influential in the decision-making process of the SNN. By utilizing ISAM, we can explore the impact of suppressing or removing specific spikes on the overall performance of the SNN.

The application of ISAM in our experiments shows promising outcomes, which motivates further investigation into its effectiveness and implications. Understanding the underlying reasons behind the observed performance improvements and the characteristics contributing to its efficacy at different layers requires more in-depth analysis.

### 2.6. Experimental setup

This section of the paper presents the details of the spiking neural network architecture and training as well as the evaluation process used in our study. In Section 2.6.1, we describe the SNN architecture and training methodologies used for image classification tasks. We also present the parameters used for training the SNNs and how the networks were trained. In Section 2.6.2, we describe the evaluation process used to evaluate the performance of the SNNs, as well as the metrics used to measure the performance of the SNNs and the XAI methods. This section provides a comprehensive overview of the experimental setup used in our study and will enable readers to reproduce the results presented in this paper.

#### 2.6.1. Spiking neural network architecture and training

We conduct all experiments on a LeNet-5 (Lecun et al., 1998) inspired convolutional network spiking-architecture to be able to compare to related work currently limited to convolutional layers. The architecture consists of 3 sets of spiking convolutional layers followed by feature extractor pooling layers and a set of classification fully connected layers.

To train the SNNs, we employed SLAYER (Shrestha and Orchard, 2018) as training-framework to access surrogate gradients from backpropagation. Two SNN were created, one for the CIFAR-10 (grayscale) dataset (Krizhevsky, 2009) with 63% validation accuracy and one for the event-based DVS128 Gesture dataset (Amir et al., 2017) with a final validation accuracy of 81.4%. DVS128 consists of gestures which have been recorded with an event-based camera, thus generating data directly in the spiking domain. Each sample has been recorded over a period of 1400ms. Please refer to Appendix 7 for details about network parameters, configuration and comparison of training results. Parameters for the baseline *post hoc* approaches LIME and RISE and SAM can be found in Appendix 8.

#### 2.6.2. Evaluation process

We conduct visual inspection and quantitative assessments on real-valued image inputs and spiking event data. First, we evaluate our end-to-end method for pixel-level attributions on CIFAR-10 comparing four types of neural coding algorithms, with differing spatio-temporal properties. Second, we use the DVS128 dataset to compute spike-attribution maps. For each dataset, a SLAYER trained Spiking-CNN was used to classify the inputs, and retrieve their gradients.

In the following, qualitative and quantitative results of selected methods will be presented, complementary results can be found in the Supplementary material.

## 3. Results

### 3.1. Visual inspection of pixel attribution maps

**Figure 4:**
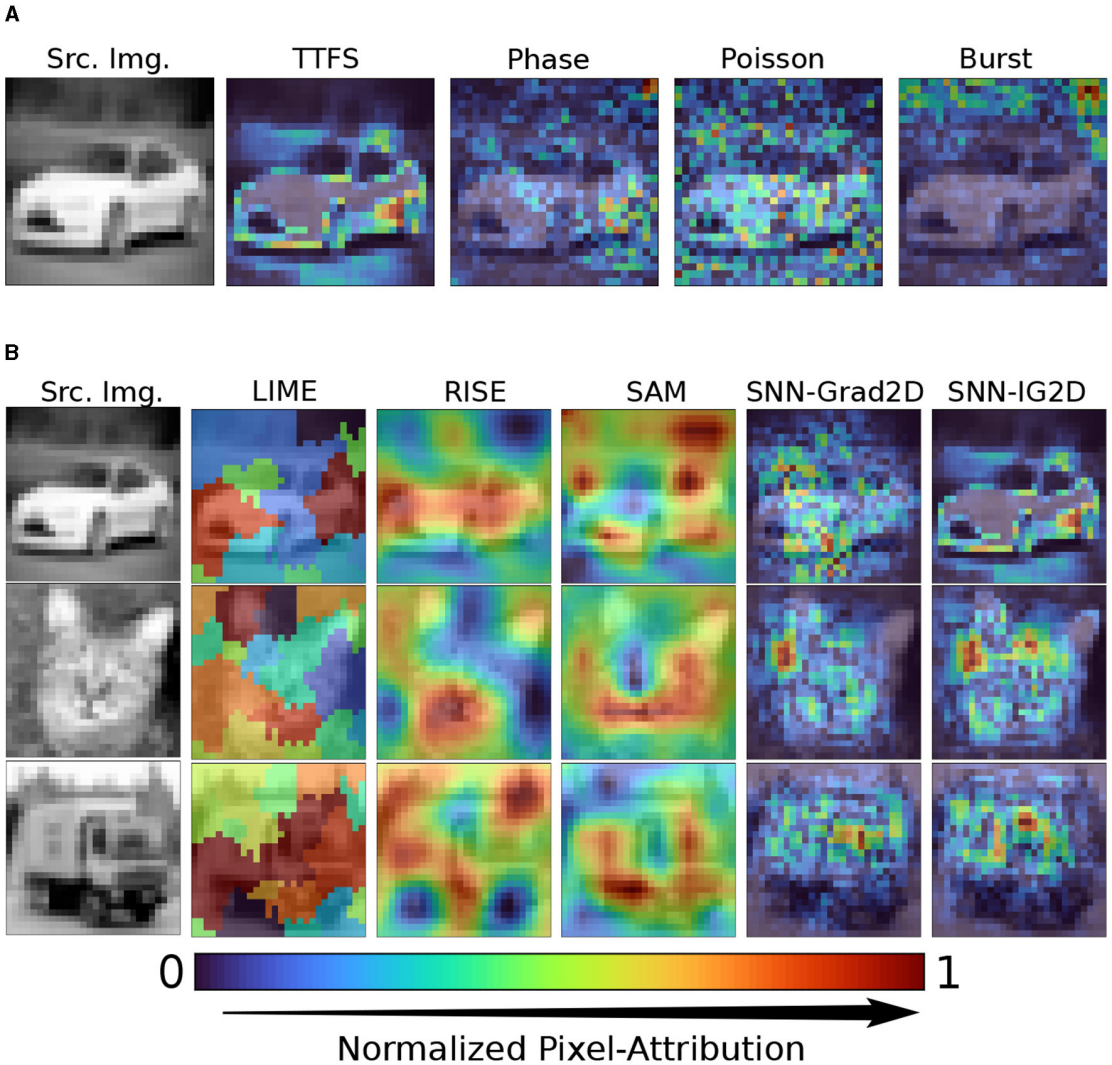
Qualitative visual inspection of 2D end-to-end pipeline attribution results. **(A)** Comparison of SNN-IG2D attribution maps on different neural encodings: Time to first spike, Phase, Poisson, and Burst coding. **(B)** Comparison to related *post hoc* attribution methods for SNNs, for TTFS encoding: model-agnostic methods (LIME, RISE), activation map visualization method (SAM) and ours (SNN-Grad2D, SNN-IG2D). SAM, SNN-Grad2D, and SNN-IG2D using summation mapping to map spike attributions to pixel-attributions. Shown visualizations have been normalized for ease of comparison.

In Figure 4A, we show the effect of different neural codings on the resulting attribution maps of our SNN-IG2D method. To aid the visualization of the most important features/pixels per image, each shown heatmap *H*^*i*^ is linearly normalized between 0 and 1 and then take the absolute values as follows: $H^{i^{\prime }}=|\frac{H^{i}-H_{min}^{i}}{H_{max}^{i}-H_{min}^{i}}|$. We see that the four neural codings using the mapping by summation result in vastly different maps. Attribution map's qualitative analysis cannot assess its correctness. Learned patterns need not be intuitive. TTFS produces the cleanest map, identifying more defined clusters than the rest.

Second, Phase coding looks to focus on similar features as TTFS but in a noisier manner, this might be partly due to the fact that Phase coding elicits more spikes, and a simple summation across time might not be as effective as for TTFS. Poisson and Burst coding generate heatmaps which are visually very noisy and don't seem to focus on the object. From here on onwards, we focus on TTFS neural coding with summation mapping for visualization and quantitative results.

In Figure 4B, we compare the resulting attribution maps from different attribution methods for SNNs. We compare two model-agnostic methods, which directly work on the 2D input and generate 2D heatmaps without intermediary spike attributions – LIME and RISE. We then compare to SAM, the latest model-specific explainability method for SNNs with convolutional layers utilizing spike-timing information. Finally the last two columns show the results obtained from our proposed gradient-based methods, SNN-Grad2D and SNN-IG2D.

As expected, LIME, RISE and SAM appear to distribute importance rather strongly to a large amount of input pixels, in contrast, SNN-Grad2D and SNN-IG2D are much more fine-granular and identify more detailed attribution scores. The reason for this is the trade-off of model agnostic models of accuracy vs. compute time, and the use of intermediate activation layers in SAM which are projected to a large area of pixels. By non-formal visual inspection, clusters of attribution seem to be more defined to abrupt changes in texture in SNN-IG2D than SNN-Grad2D.

### 3.2. Quantitative assessment

The average latency overhead to compute 2D attribution maps for 100 samples of the CIFAR-10 image with SNN-Grad2D is ~26 ms, SNN-IG2D (n=50) ~1.54 s, SAM ~8.39 s, RISE ~13.27 s and LIME ~49.78 s. See Table 1 for measurements on CIFAR-10, F-MNIST and MNIST using TTFS encoding.

**Table 1:** Average measured latency [in seconds] (SD) to compute an attribution map on 100 sampled images on a PC with a Core i9-10980XE CPU and GeForce RTX 3090 GPU—Lower is better.

	**SNN-Grad2D**	**SNN-IG2D**	**SAM**	**RISE**	**LIME**
C-10 (TTFS)	0.026 (0.001)	1.54 (0.04)	8.39 (0.17)	13.27 (0.37)	49.78 (0.67)
F-MNIST (TTFS)	0.026 (0.001)	1.54 (0.03)	9.23 (0.15)	14.81 (0.57)	51.94 (0.66)
MNIST (TTFS)	0.027 (0.001)	1.52 (0.04)	9.14 (0.11)	15.87 (0.20)	56.18 (0.22)

#### 3.2.1. Deletion and insertion scores for 2D attribution methods

Perturbation of high scoring features should cause a higher output variation. In Deletion (Insertion) experiments, as used in Samek et al. (2017) and Ancona et al. (2017), the *k* most important features—spikes or pixels—based on the attribution map are removed (inserted), and the degradation (improvement) of the classification score and class recall accuracy are iteratively recorded. We perform these perturbation experiments on a subset of 600 samples of the validation data sets that the network correctly classified, to avoid a doubtful correctness evaluation of an attribution map obtained from wrong predictions. Perturbation of features means changing it to some predetermined value (the *baseline*). The choice of baseline is an important factor to keep in mind when evaluating attribution methods, as it can create some artifacts, which confuse the models as seen in Samek et al. (2017) and Ancona et al. (2017). To reduce the effect of the artifacting by the baseline choice, we evaluate our method with both Deletion and Insertion strategies. In Deletion pixels are iteratively replaced with three baselines: black, gray, and white, while for spikes the baseline is no spike. For Insertion the starting point is the corresponding baseline.

**Figure 5:**
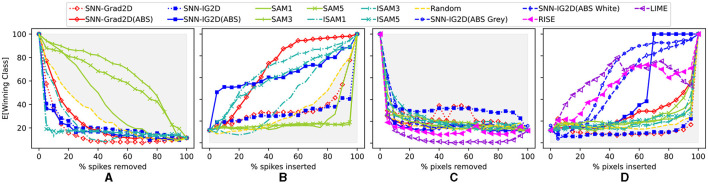
Quantitative results on feature attribution maps based on spike and pixel perturbations. X axis: Percentage of input perturbations. Y axis: Average accuracy of the SNN (using TTFS on 600 correctly identified images in the original CIFAR-10 dataset). **(A, B)** Spike perturbations: Deletion respectively insertion of *k%* most salient input spikes (after input image encoding) comparing: SNN-Grad2D, SNN-IG2D, SAM(layers:1,3,5), I-SAM(layers:1,3,5), and Random spike-perturbations as baseline. **(C, D)** Pixel perturbations: The pixel-level 2D attribution map scores obtained after *summation* mapping are used to perturb the input image to measure their efficacy to detect the most important input pixels; comparing: SNN-Grad2D, SNN-IG2D, I-SAM, LIME, RISE, and Random spike-perturbations as baseline. Gray shaded area highlights performance worse than random. See Figure 7 and Supplementary material S13 for individual plots with error bars.

In Figure 5, we assess the performance of our 2D SNN attribution methods. As the 2D pipeline produces two attribution maps (first in spikes, then in pixels) we apply the perturbation experiments at both stages. Figures 5A, B measures the perturbation of the event stream (after the image encoding). Here, we compare our methods SNN-Grad2D and SNN-IG2D to SAM (at different convolutional layers) and random perturbation as baseline. Our gradient-based methods produce positive or negative attribution scores. We evaluate our methods with both the raw score, or with the absolute value (postfix ABS) as commonly done in the equivalent XAI related work for ANNs.

##### 3.2.1.1. Interpretation of perturbation results for encoded event stream

In spike perturbations (Figures 5A, B), SAM (at all layers) performs worse than random. SAM captures neuronal activity at convolutional layers, but results indicate that this activity map does not directly translate to a true input attribution map. This matches our expectation of the non-linear relationship between activation and attribution. We decided to evaluate the inverted version ISAM (the negative of SAM scores), and its performance surpassed the random baseline. Our method SNN-IG2D(ABS) performs better than SAM in both perturbation experiments. Remarkably, inverse SAM at layers 3 and 5 do better in spike removal and competitively in spike insertion. We speculate that the SAM attention maps are in this case indicative of a dominant sensory suppression behavior (Kim and Panda, 2021). In spike insertion, SNN-IG2D(ABS) quickly identifies important features, which rapidly, with about 5% of spikes, increase the SNNs performance to around 50% accuracy in classifying the data. At around 30% insertion, SNN-Grad2D(ABS) and ISAM at layer 3 overtake SNN-IG2D(ABS). The reason behind this behavior and why ISAM at layer 3 performs better than a later or an earlier layer is not clear to us.

Next, Figures 5C, D measure the perturbation results of 2D pixel data (before encoding). We compare again SNN-Grad2D, SNN-IG2D (on three baselines) and SAM, ISAM using summation mapping to obtain 2D attribution maps, as well as the model-agnostic methods RISE (Petsiuk et al., 2018) and LIME (Ribeiro et al., 2016).

##### 3.2.1.2. Interpretation of perturbation results for input pixel data

In pixel perturbations, the model agnostic methods are capable to identify important features quite well and justify their high computation cost. Both SAM and ISAM perform worst than random in pixel removal and better than random in pixel insertion. Again, we believe this is due to activation maps not representing good attribution maps. Our method SNN-IG2D(ABS) performs the best among the model-specific methods. It is possible that our methods did not outperform model-agnostic ones in part due to the simplistic mapping across the time dimension. For pixel removal, the black baseline produces better results, while in pixel insertion a gray baseline is better. This can intuitively understood: removing a pixel with the lowest value (black) is a stronger perturbation than “removing it” with a bright value (white). Similarly, inserting a few important features in a completely empty (black) background is a significant deviation from the training data distribution, thus a gray baseline recreates a closer sample of the training distribution.

#### 3.2.2. Deletion and insertion scores for 3D spike-attribution methods

We evaluate our SNN-Grad3D and SNN-IG3D methods on event-based data, using the same perturbation strategy.

##### 3.2.2.1. Interpretation of perturbation results for native event stream

**Figure 6:**
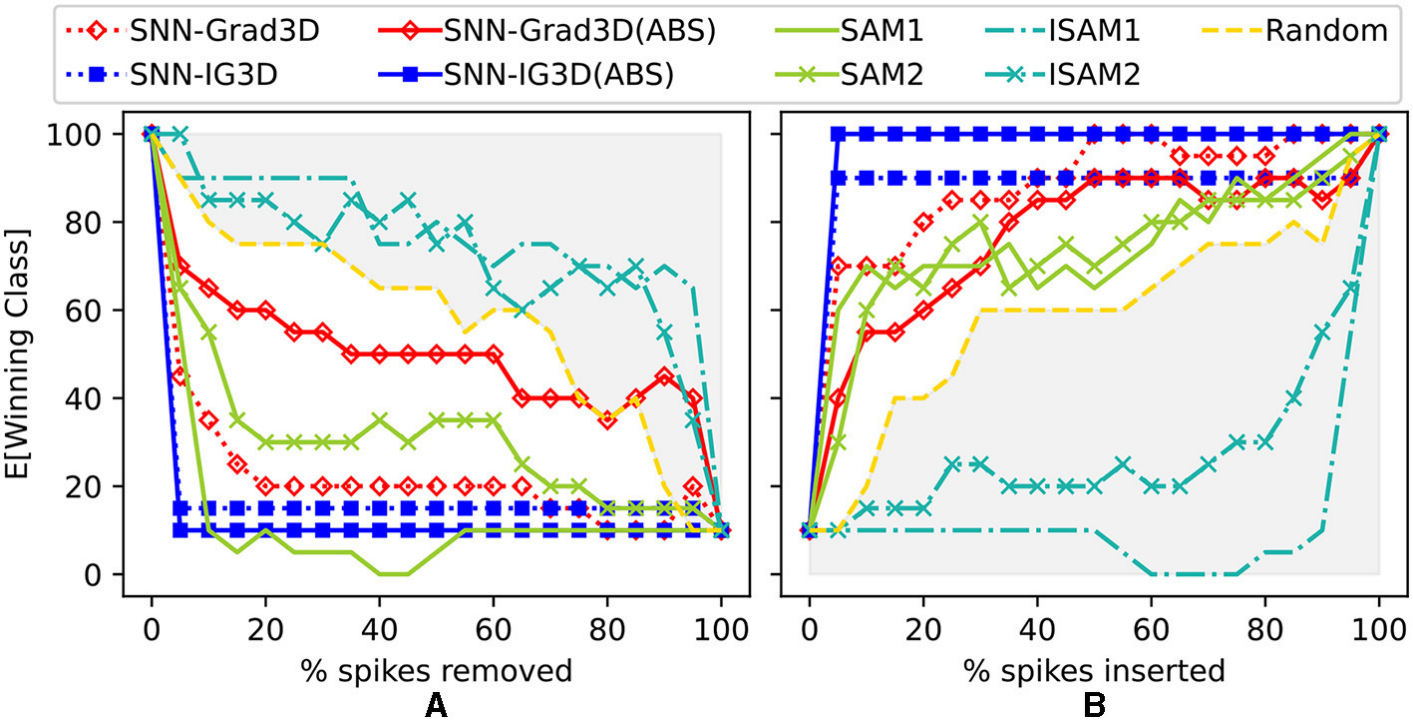
Spike-perturbation experiment (spike removal: **A** and spike insertion: **B**) on SNN trained on event-based DVS128 Gesture dataset. Comparison of SAM(layers 1&2), I-SAM(layers 1&2), SNN-Grad3D, and SNN-IG3D. Additionally, compared to a Random perturbation baseline (yellow, dashed). Gray shaded area highlights performance worse than random.

**Figure 7:**
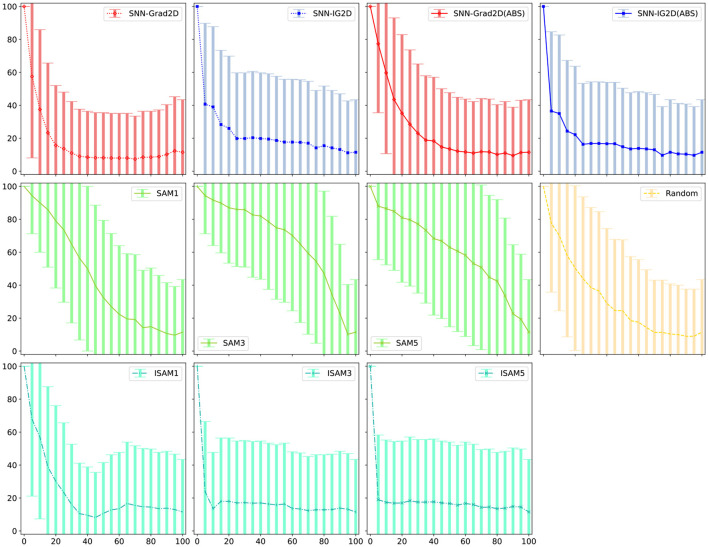
Error bars for Figure 5A—% spikes removed vs. E[Winning Class]. Error bars for the other experiments can be found in the Supplementary material.

As seen in Figure 6, SNN-IG3D, using the spike sampling (ii) of Appendix 10, vastly achieves to identify most important spikes quickly: with deletion (or insertion) of less than 5% of the potential spike-locations resulting in total degradation (restoration) of the original winning class expectation. To note, inversely to the encoded image dataset, the sparse and temporal properties of this DVS128 Gesture dataset seem to more naturally fit with SAM method. The original SAM method now is better than the inverse ISAM at all layers. In the previous experiment, as better appreciated in Figure 7, it can be seen that ISAM was better. Also interestingly, and arguably due to the aforementioned nature of activation maps, there is no guideline as to which layer is most useful. Here, in spike removal layer 1 (earlier) is clearly superior to layer 2 while in the image experiments, sometimes the outer layers were better. Finally, the signed or absolute value version of our methods also result in different performances. Both SNN-IG2D and SNN-IG3D get better results in all out experiments in the ABS case. The SNN-Grad3D signed version is better for both insertion and deletion and SNN-Grad2D is significantly improved by taking the absolute value in all but the spike removal experiment, where it moderately decreases. This behavior seem to be related to the difference of information carried by each method. Gradients capture very local behavior, so big local negative change is not necessarily the same as indicating that the pixel is not a positive influence.

## 4. Conclusions and future work

Explainability of SNNs poses unique challenges, requiring specialized methods to understand their decision-making processes. In this paper, we have investigated the effectiveness of gradient-based attribution methods for SNNs trained with the surrogate backpropagation approach.

Our findings indicate that gradient-based methods, namely SNN-Grad2D, SNN-Grad3D, SNN-IG2D, and SNN-IG3D, offer several advantages over existing approaches. Firstly, these methods require significantly less computation time compared to other techniques. Secondly, they are not limited to convolutional operations as methods producing activation maps, which enhances their applicability to various neural coding schemes. Thirdly, unlike deconvolutional methods, they do not necessitate training an extension of the model. Lastly, the perturbation experiments demonstrate that these methods provide true attribution scores.

While discussing the preference between Integrated Gradients and Gradient methods, it is worth noting that SNN-IG has shown particular superiority. SNN-IG excels in providing finer-grained attribution maps compared to model-agnostic or activation-based methods for SNNs. The integration of gradients along the input space, as employed by SNN-IG, enables a more comprehensive understanding of the influence of different input features on the SNN's decisions.

In future work, we envision several avenues for enhancing Explainable Neuromorphic Computing. Firstly, it would be valuable to refine the surrogate-gradient approach to mitigate inaccuracies that may impact gradient-based methods. This would further improve the reliability and interpretability of the attribution maps generated. Secondly, exploring the adaptation of other gradient-based eXplainable Artificial Intelligence methods from ANNs to SNNs could provide valuable insights and potential advancements. Lastly, developing specific mapping techniques based on neural codings to accurately map 3D attributions back to the pre-encoded source data would enhance the interpretability of SNNs operating on event-based inputs.

Overall, this study contributes to the understanding of explainability in SNNs and opens up avenues for future research, bridging the gap between neuromorphic computing and interpretable artificial intelligence.

## Data availability statement

The original contributions presented in the study are included in the article/Supplementary material, further inquiries can be directed to the corresponding author.

## Author contributions

AB and RR contributed to conception and design of the study and wrote sections of the manuscript. AB organized the database, performed the statistical analysis, and wrote the first draft of the manuscript. All authors contributed to manuscript revision, read, and approved the submitted version.
