# Supplementary material for: Gradient-based feature-attribution explainability methods for spiking neural networks
Source: Front Neurosci. 2023 Sep 27;17:1153999. doi: 10.3389/fnins.2023.1153999 (PMC10565802; doi:10.3389/fnins.2023.1153999)
Supplement: Supplementary file 1 [file Data_Sheet_1.PDF]

Supplementary Material

Number of words in Supplementary: 2173
Number of figures in Supplementary: 8
Number of tables in Supplementary: 9

1 EXTENDED QUALITATIVE VISUALIZATION OF 2D ATTRIBUTION MAPS

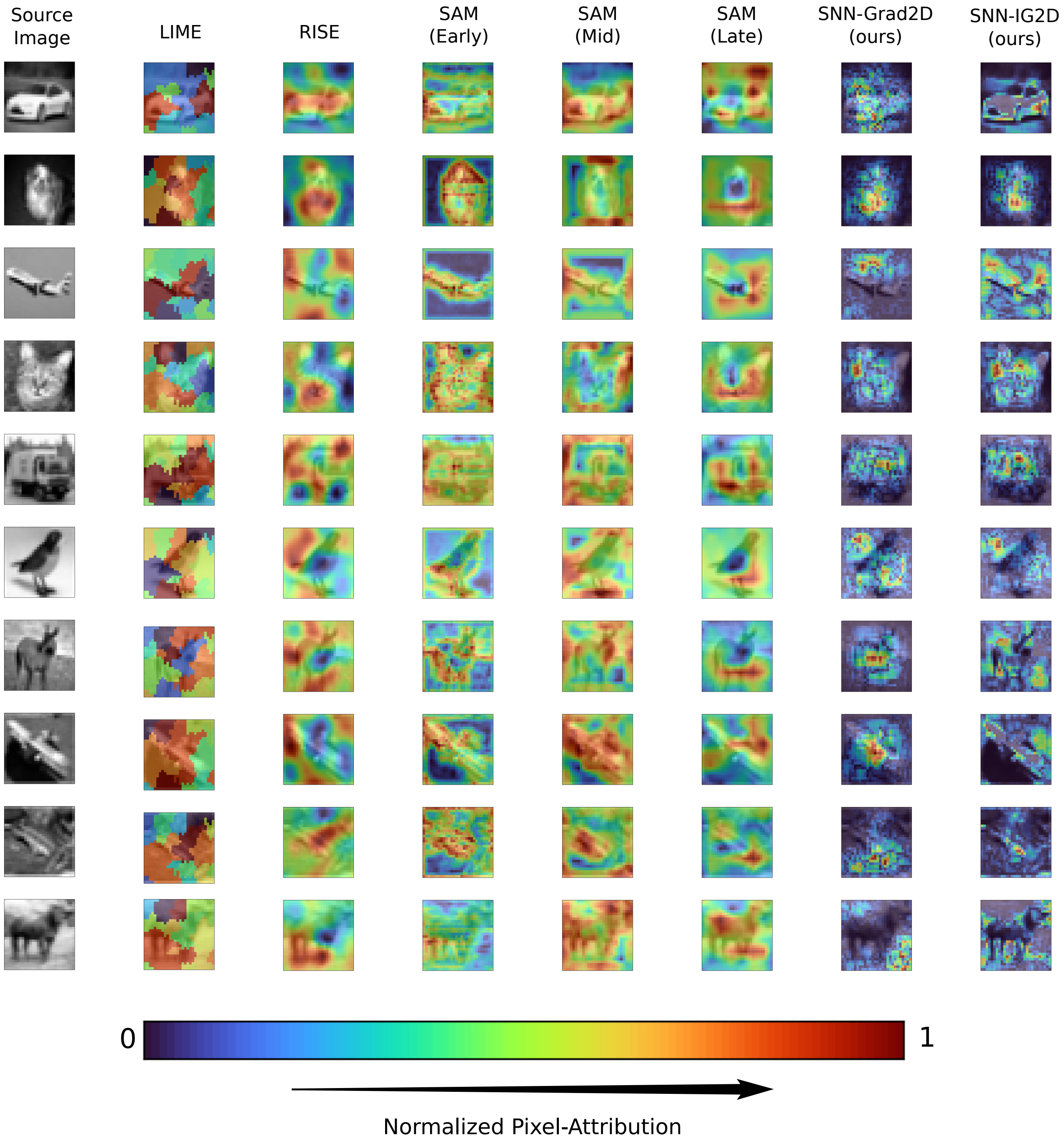

Figure S1. Sample Attribution Maps for each class in the CIFAR-10 dataset. Each pixel show the absolute value of the attribution scores normalized between 0 (blue) and 1 (red). For SAM, the activation maps are shown for three different intermediate convolutional layers.

2 LATENCY RESULTS

The latency of the proposed gradient-based attribution methods compared to alternative approaches (with the parameters described in Appendix 8) are shown in Table S1.

	SNN-Grad2D	SNN-IG2D	SAM	RISE	LIME
C-10 (TTFS)	0.026 (0.001)	1.54 (0.04)	8.39 (0.17)	13.27 (0.37)	49.78 (0.67)
F-MNIST (TTFS)	0.026 (0.001)	1.54 (0.03)	9.23 (0.15)	14.81 (0.57)	51.94 (0.66)
MNIST (TTFS)	0.027 (0.001)	1.52 (0.04)	9.14 (0.11)	15.87 (0.20)	56.18 (0.22)

Table S1. Average measured latency [in seconds] (SD) to compute an attribution map on 100 sampled images on a PC with a Core i9-10980XE CPU and GeForce RTX 3090 GPU –Lower is better

3 NEURON MODEL

Various spiking neuron models have been proposed in the literature exhibiting the properties of memory through their internal state, and non-differentiable spiking function. Most developed spiking neuron models are the Spike Response Model (SRM) (Gerstner, 1995; Gerstner et al., 2014) and the Leaky Integrate-and-Fire (LIF) model (Gerstner et al., 2014), which is a special case of SRM. In this work, we use the SRM type of spiking neuron provided by `SLAYER` (Shrestha and Orchard, 2018), for which incoming spikes are converted into a spike response signal, $a_i(t)$ by convolving $s_i(t)$, the neurons' output spikes, with the spike response kernel $\epsilon(\cdot)$, as such $a_i(t) = (\epsilon * s_i)(t)$. The refractory response of the spiking neuron – which defines the reduced neural response to incoming spikes shortly after spiking – can be represented similarly as: $(v * s)(t)$, where $v(\cdot)$ is the refractory kernel.

Spike response signals are then scaled by the synaptic weights w_i, which gives us the Post Synaptic Potential (PSP). Finally, the neurons' membrane potential, $u(t)$, is the sum of all `PSPs` and refractory responses:

$$u(t) = \sum w_i(\epsilon * s_i)(t) + (v * s)(t) = w^T a(t) + (v * s)(t) \tag{S1}$$

The SNNs spiking function (i.e. its activation function) is governed by a thresholding mechanism; when $u(t)$ reaches the threshold θ, the neuron generates a spike and resets it's membrane potential to the resting potential (0). More formally, the spiking function can be written as Equation S2:

$$s(t) = \begin{cases} 1, & \text{if } u(t) \geq \theta, \\ 0, & \text{otherwise.} \end{cases} \tag{S2}$$

4 ADDITIONAL NEURAL CODES

4.1 Time to First Spike Coding:

For every pixel $p_{m,n}$ of an input image of size $M \times N$, with $0 \geq m \geq M$ and $0 \geq n \geq N$, generate a spike train $S_{p_{m,n}}$ of length T timesteps. TTFS produces a single spike per spike train, the timing of the spike is equal to the pixel value. As such, we can compute the occurence of a spike at timestep t for spike

train $S_{p_{m,n}}$ as follows:

$$S_{p_{m,n}}(t) = \begin{cases} 1, & \text{if } t = p_{m,n}, \\ 0, & \text{otherwise.} \end{cases} \tag{S3}$$

4.2 Poisson Coding:

is a form of rate coding in which the analog value is converted to a spike train using a Poisson generator function with a rate proportional to the input pixel value. Basically, the Poisson spike generator is implemented by comparing input pixel values normalized between 0 and 1; $\hat{p}_{m,n} = \frac{p_{m,n} - min(P)}{max(P) - min(P)}$ with a random number $X_{m,n,t}$ sampled from a uniform distribution between 0 and 1, $X \sim U(0,1)$, where $p_{m,n}$ is the pixel at (m,n), $\hat{p}_{m,n}$ is the normalized pixel value, and P is the set of possible pixel values, here ranging from 0 to 255. At each timestep if a normalized pixel value $\hat{p}_{m,n} \geq X_{m,n,t}$, a spike is generated at that spatiotemporal location ($S_{p_{m,n,t}}$).

$$\begin{aligned} X &\sim U(0,1), \\ \hat{p}_{m,n} &= \frac{p_{m,n} - min(P)}{max(P) - min(P)} \\ S_{p_{m,n}}(t) &= \begin{cases} 1, & \text{if } \hat{p}_{m,n} \geq X_{m,n,t}, \\ 0, & \text{otherwise.} \end{cases} \end{aligned} \tag{S4}$$

5 BURST CODING:

inspired by the short bursts of spikes in the brain, this scheme encodes the information in the `Inter-Spike-Intervals` (ISI) – the timing between spikes. Biological observations have shown that neurons in the brain communicate through short bursts of spikes, rather than long-continuous spike-trains. This encoding is another increase in spatial and temporal complexity, as information resides in the timings between spikes. (**?**) define burst coding as follows: First, normalize the input pixel between 0 and 1: $\hat{p}_{m,n} = \frac{p_{m,n} - min(P)}{max(P) - min(P)}$, with *P* being the set of possible pixel values, here ranging from 0 to 255. Then compute the number of spikes:

$$N_s(p_{m,n}) = \lceil N_{max} * \hat{p}_{m,n} \rceil \tag{S5}$$

Where $\lceil \cdot \rceil$ defines the ceiling function, N_{max} the maximum number of spikes and P the normalized pixel value. Then, the ISI is computed:

$$\text{ISI}(p_{m,n}) = \begin{cases} \lceil -(T_{max} - T_{min})\hat{p}_{m,n} + T_{max} \rceil, & \text{if } N_s > 1, \\ T_{max}, & \text{otherwise.} \end{cases} \tag{S6}$$

where T_{min} and T_{max} are the minimum and maximum interval values that the ISI can take. Thus, dictating a larger or smaller amount of spikes in the burst window. Similarly as (**?**), we use a N_{max} value of 5. For T_{min} and T_{max} we chose 2 timesteps and 10 timesteps respectively.

6 PHASE CODING:

(**?**) proposed a simple phase coding scheme by converting input pixels into their binary representation. The bit "*1*" signals a spike. A single phase covers 8 timesteps, which is determined by the binary representation of the largest pixel intensity, 255 which corresponds to one byte or 8 bit. The resulting binary representation of the pixel value is then used as spike train, starting at timestep 0.

7 NETWORK CONFIGURATIONS DETAILS

7.1 CIFAR-10 Net - SLAYER Neuron Model Training Parameters

Table S2 presents the neuron parameters used to train the CIFAR-10 networks for all different neural codings.

Architectures for all experiments were trained using the Adam optimizer (Kingma and Ba, 2015) with a learning rate of 0.01, beta-coefficients of $(0.9, 0.999)$ and ϵ numerical stability parameter of 10^{-8}. These parameters correspond to the default suggested by (Kingma and Ba, 2015). All architectures trained on CIFAR-10 (grayscale) were trained for 200 epochs, on the respective neural codings. Datasets were split into 80% test-set and 20% train-set.

SLAYER Parameter	Value
Neuron Type	'SRM'
Simulation Time	300
Theta	10
tauSr	10.0
tauRef	1.0
scaleRef	2
tauRho	1
scaleRho	1

Table S2. SLAYER Parameters used to train CIFAR-10 networks

7.2 128DVS Gesture Net - SLAYER Neuron Model Training Parameters

Table S3 presents the neuron parameters used to train the CIFAR-10 networks for all different neural codings.

Architectures for all experiments were trained using the Adam optimizer (Kingma and Ba, 2015) with a learning rate of 0.01, beta-coefficients of $(0.9, 0.999)$ and ϵ numerical stability parameter of 10^{-8}. These parameters correspond to the default suggested by (Kingma and Ba, 2015). The architecture trained on the Gesture Dataset was originally trained for 200 epochs. Datasets were split into 80% test-set and 20% train-set.

SLAYER Parameter	Value
Neuron Type	'LOIHI'
Simulation Time	1450
vThMant	80
vDecay	128
iDecay	1024
refDelay	1
tauRho	1
scaleRho	1

Table S3. SLAYER Parameters used to train DVS128Gesture network

7.3 Appendix: Tabular Training SNN Training Results

The following tables present the training and validation accuracies after training each network for 200 epochs for each dataset and neural coding combination.

CIFAR-10 (gray)

Coding	Test Acc.	Val. Acc
TTFS	50.1	48.3
Phase	68.3	51.2
Burst	58.1	49.1
Poisson	60.0	41.2

Table S4. Testing and validation accuracies of SNNs trained on CIFAR10 (grayscale) for 200 epochs

DVS Gesture

Data Type	Test Acc.	Val. Acc
Event-Based	97.6	81.4

Table S5. Testing and validation accuracies of SNNs trained on DVS128 Gesture for 200 epochs, parameters and network configuration from (Shrestha and Orchard, 2018)

8 PARAMETERS FOR RELATED WORK COMPARISON

In this appendix section, we lay out the chosen parameters for state-of-the-art methods we compare to. Results for comparison were taken by running the publicly available code and parameters adapted to our setup. Parameters were chosen based on the methods' original paper recommendations, if not the case, we explicitly state it here.

SNN-IG Parameter	Value
n_steps	50

Table S6. IG-based: SNN-IG3D and SNN-IG2D Image Explainers Parameters

LIME Parameter	Value
num_features	1000
segmenter	'quickshift'
kernel_size	1
max_dis	200
ratio	0.2
num_samples	10000
top_labels	10
min_weight	0.01

Table S7. LIME Image Explainer Parameters

RISE Parameter	Value
n_masks	2000
s (size)	8
p1	0.5

Table S8. RISE Image Explainer Parameters

SAM Parameter	Value
p (trail)	5ts
gamma	0.5

Table S9. SAM Parameters

9 MAPPING FUNCTIONS FROM SPIKE-ATTRIBUTION MAPS TO REAL-VALUED ATTRIBUTION MAPS

To close the explanation-loop for the real-value input data, a mapping from the spike-level attribution-box to the original input space is necessary. For 2D image data, this corresponds to a mapping from a 3D attribution-box to a 2D attribution heatmap. In this work, we explored various basic mappings to aggregate the gradients across the three dimensional space (height, width and time) to the two dimensional space (height, width).

This is not trivial, as the initial encoding process is not part of the SNNs training, thus, the SNNs never learns what a *valid*[1] spike-train is. Thus, the real-valued gradient-box does not show any inherent relation to the encoding, disallowing a simple decoding through an inverse function.

We experimented with these basic mappings to aggregate the gradients across the three dimensional space (Height, Width and Time) to the two dimensional space (Height and Width):

1. **Summation**: Gradients are summed across the time-axis, refer to Equation S7.

[1] In this context, a *valid* encoding means a spike-train representing an input pixel correctly.

2. **Maximum gradient per coordinate**: Each spatial coordinate maps the highest gradient to the 2D space, refer to Equation S8.
3. **Average gradients across timesteps**: Gradients are averaged over time, see Equation S9.

$$S_{f_{m,n}}(x) = \sum_{t=0}^{t_{max}} S_{f_{m,n,t}}(x) \tag{S7}$$

$$S_{f_{m,n}}(x) = \max_{x_{i,j,0},\ldots x_{m,n,t_{max}}} (S_{f_{m,n,t}}(x)) \tag{S8}$$

$$S_{f_{m,n}}(x) = \frac{\sum_{t=0}^{t_{max}} S_{f_{m,n,t}}(x)}{t_{max}} \tag{S9}$$

10 SNN-IG3D EVENT STREAM PATH APPROACH.

The intuition behind the original `Integrated Gradients`, is to accumulate a pixels local gradients and attribute its importance as a score for how much it adds or subtracts to the model's overall output class probability. To do so, small steps are interpolated along a straight line in the feature space between a baseline and the original pixel value. Event streams are binary timeseries, we cannot create a path from a fully empty baseline to the original data, as the all-or-nothing nature of spikes disallows them from having floating point values. To circumvent that and create a path from this empty baseline (an all-zeros timeseries) to the original event stream (ones and zeros), we aim to reproduce the original intuition by iteratively sampling from the time dimension, as to approach the interpolation approach over the time dimension.

There are multiple ways possible to take these steps along the time dimension feature space (of T timesteps), we explored two deterministic sampling methods:

(i) First is done by a divide-and-conquer strategy, where we start by sampling the events from the timestep in the middle of the timewindow (i.e.: $\frac{T}{2}$). We then iteratively add timesteps by taking the timestep in the middle of both resulting sides, and so on until having sampled the full sample.

(ii) We select the number of interpolation steps to produce I event-streams ($I < T$) to create a path from the empty baseline to the original spike train. At each i_{th} event-stream, we select $T \times \frac{i}{I}$ timesteps (equally distanced). All spikes in the spatial dimension of the original input spike train are then included at the selected timesteps of each event-stream.

Finally, as in the original integrated gradients, we approximate the integral between the baseline and input by accumulating (cumulative average) these local gradients.

The sampling approach (ii) provided more monotonicity in the perturbation experiments. This could be due to the preference of this heuristic to sample more evenly across all time windows which may be a better match for the input expectation of the SNN.

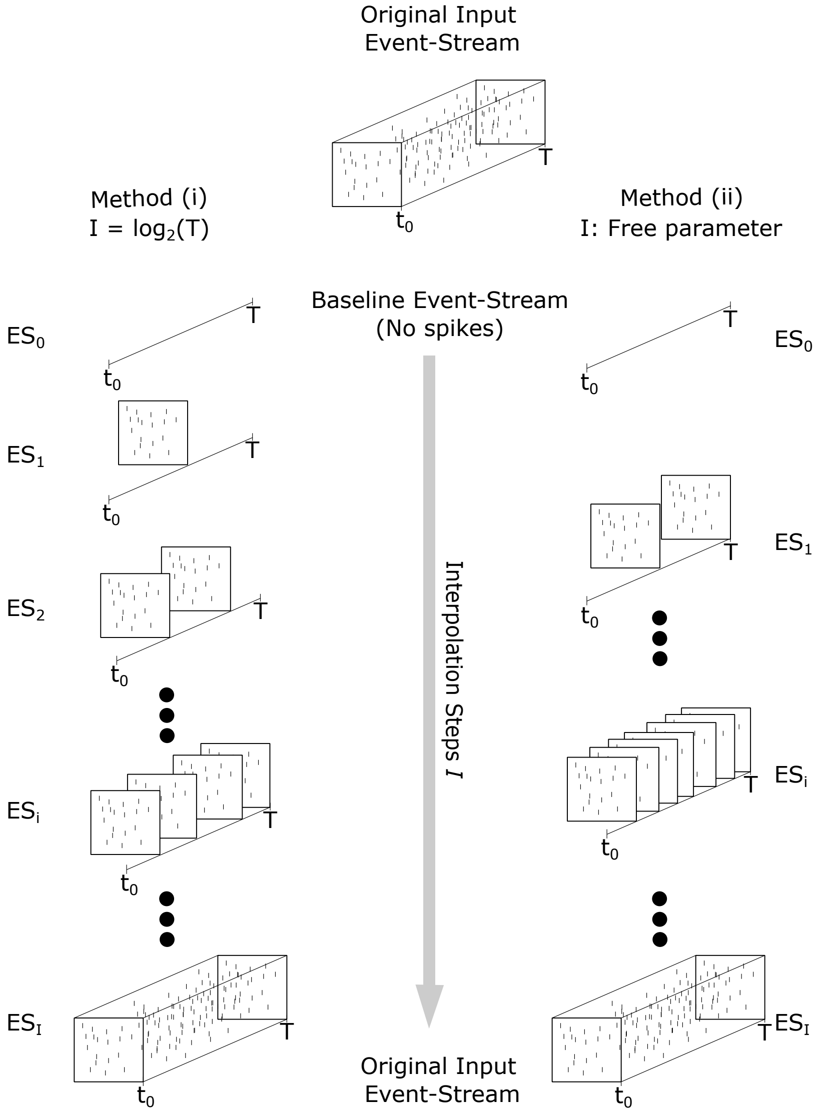

Figure S2. Visualization of explored event-stream interpolation methods for SNN-IG3D.

11 SURROGATE GRADIENT-BASED TRAINING: SLAYER

`SLAYER`(Shrestha and Orchard, 2018) is a framework built on top of pyTorch for training SNNs using a surrogate gradient and error reassignment through time. It implements various operations, such as spiking convolutions, fully connected spiking layers and more. For the backward pass, as the spiking function is non-differentiable, we use `SLAYERs` implementation of the surrogate function: $g(V) = \frac{1}{\alpha}exp(-\beta|V - V_{th}|)$, which approximates the derivative of the `Heaviside Step Function` (Spiking function). Other possible surrogate functions that have been used in research for SNN training: `SuperSpike` (Zenke and Ganguli, 2018), the derivative of a fast sigmoid function, derivative of a standard sigmoid function (Zenke and Ganguli, 2018; Zenke and Vogels, 2021) and a piece-wise linear function (Bellec et al., 2018; Zenke and Vogels, 2021).

12 PERTURBATION EXPERIMENTS—SUPPLEMENTARY TABULAR RESULTS

Quantitative results in tabular form, for the DVS128 Gesture Dataset. From the perturbation (deletion and insertion) experiments, we compute the area under the curve (AUC) to further quantify the expressive power of the methods and compare to the results of ANN's attribution methods.

	SAM(Last)	I-SAM(Last)	SNN-Grad3D(ABS)	SNN-IG3D(ABS)	Random
DVS128 Gesture	0.31	0.73	0.5	**0.12**	0.58

Table S10. Deletion of Spikes–Smaller is better

13 SUPPLEMENTARY RESULTS OF PERTURBATION EXPERIMENTS

Individual plots of the insertion and deletion experiments of Figures 3 and 4 are shown here including the error bars.

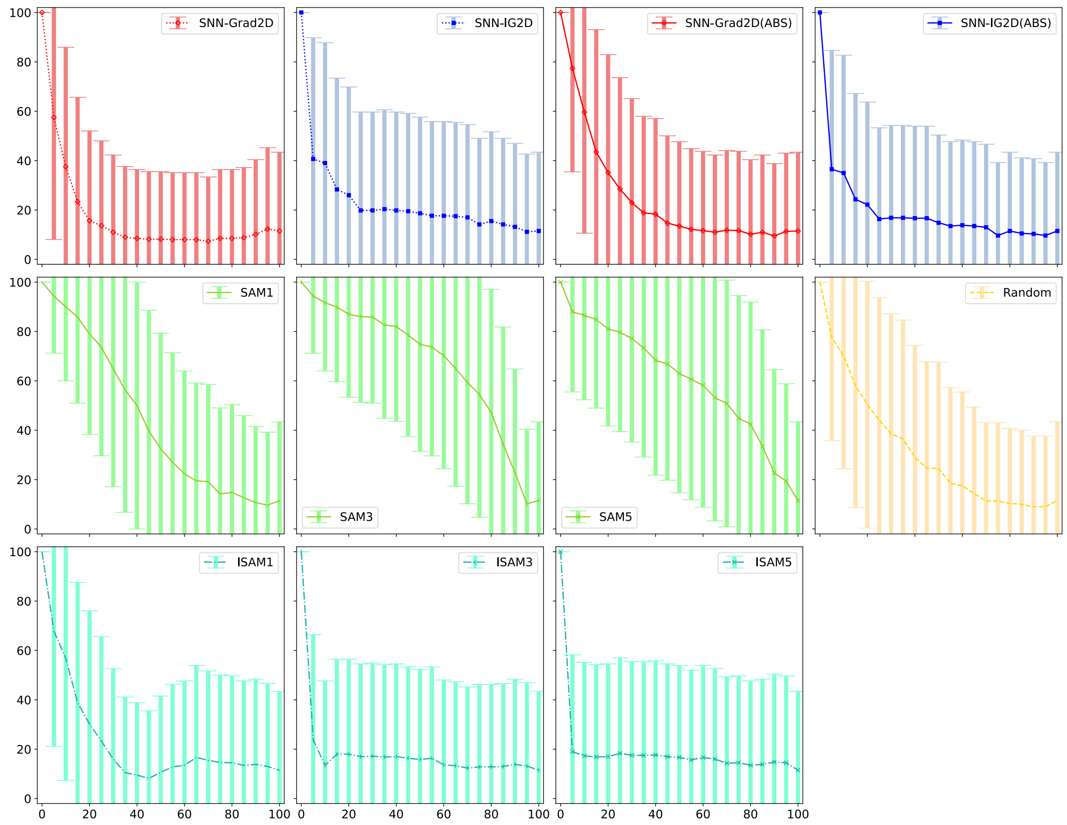

Figure S3. Error bars for Figure 3 (a) - % spikes removed vs E[Winning Class]

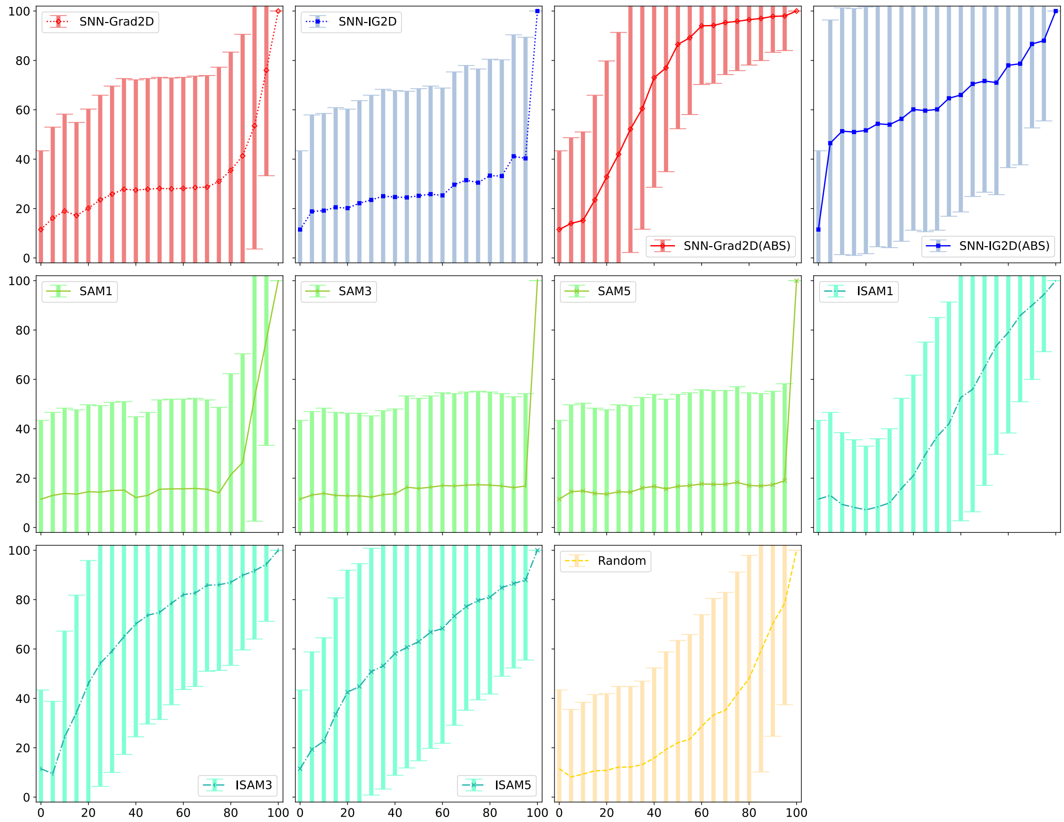

Figure S4. Error bars for Figure 3 (b) - % spikes inserted vs E[Winning Class]

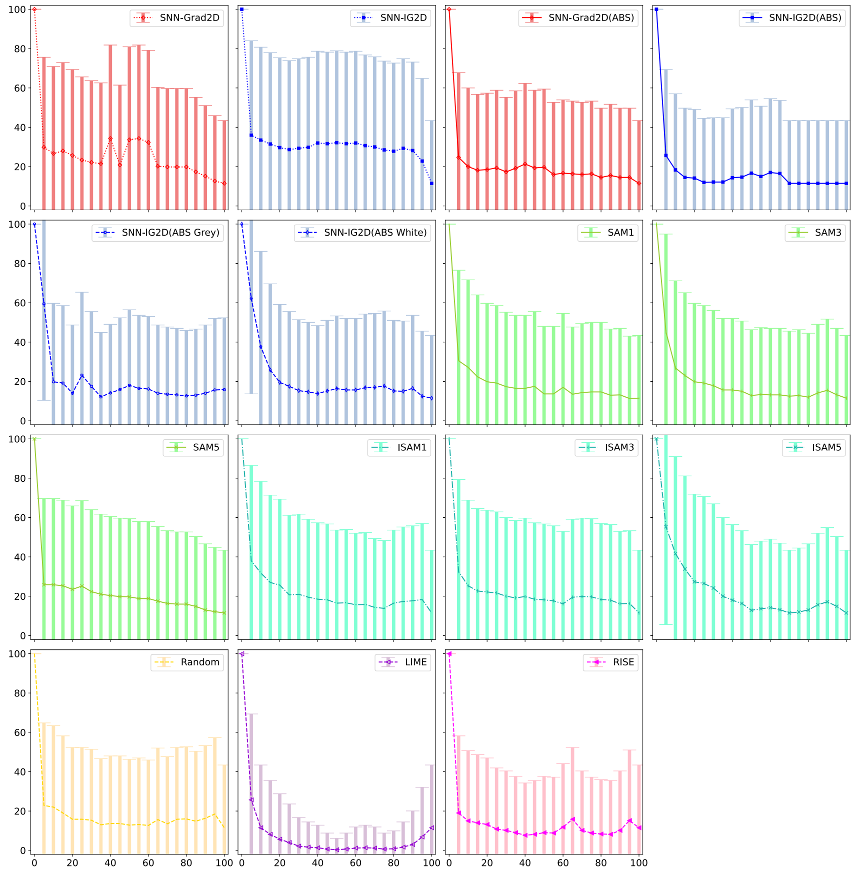

Figure S5. Error bars for Figure 3 (c) - % pixels removed vs E[Winning Class]

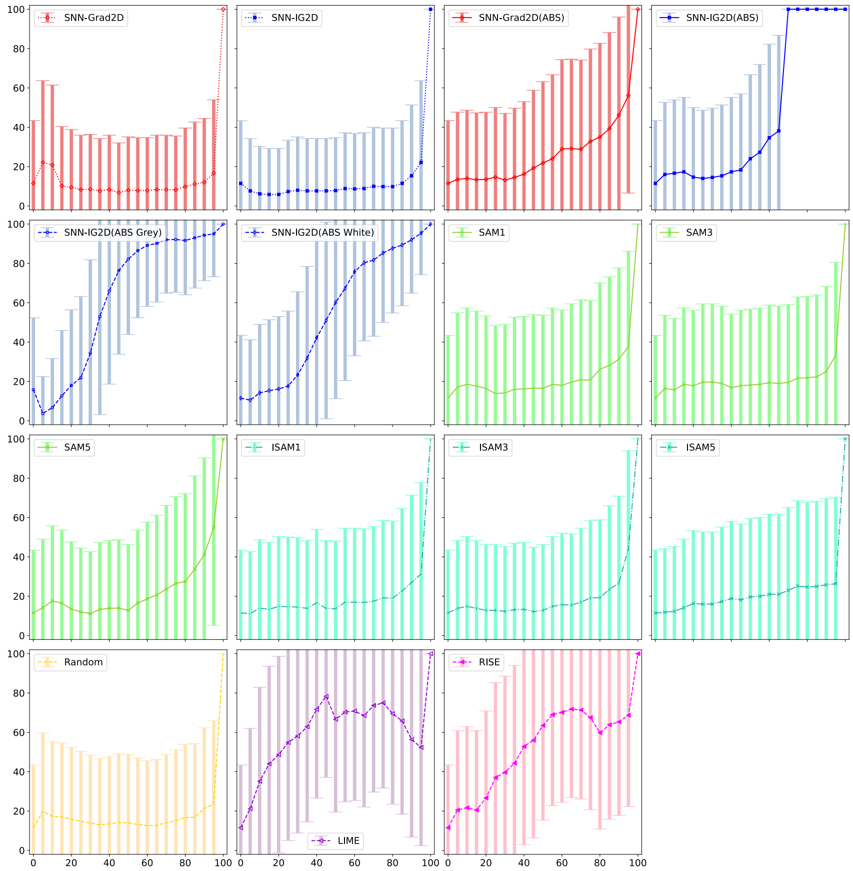

Figure S6. Error bars for Figure 3 (d) - % pixels inserted vs E[Winning Class]

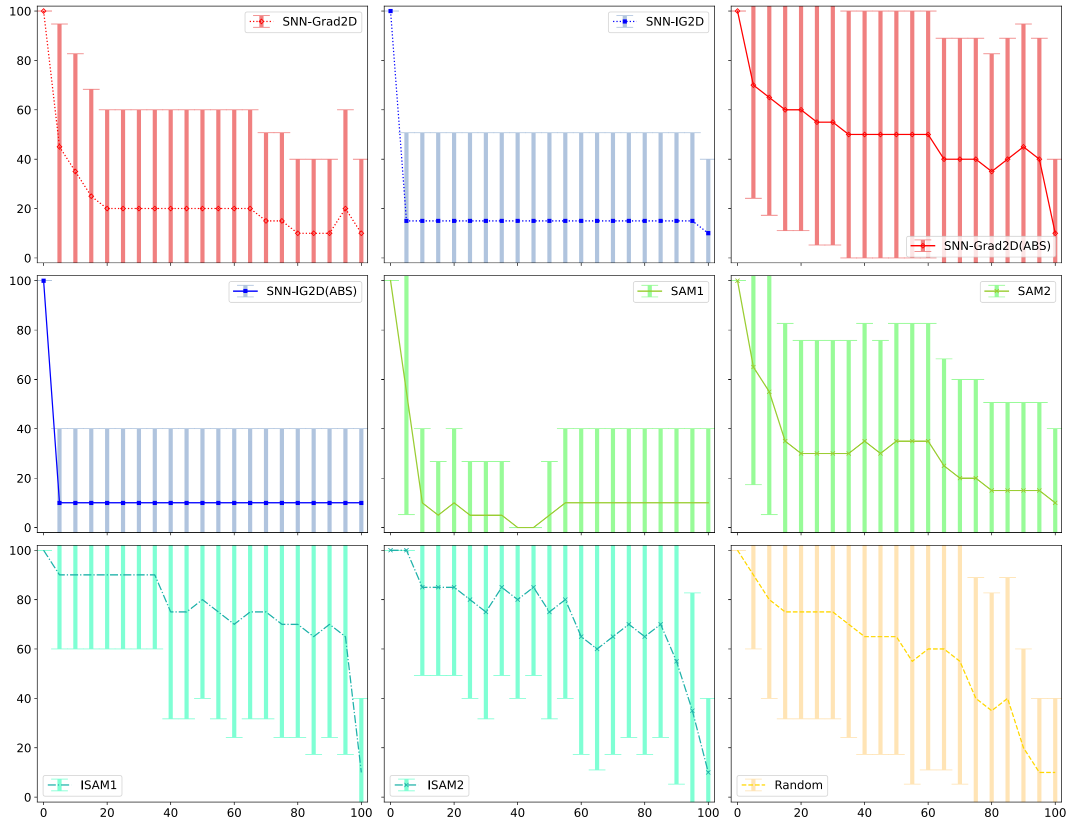

Figure S7. Error bars for Figure 4 (a) - % spikes removed vs E[Winning Class]

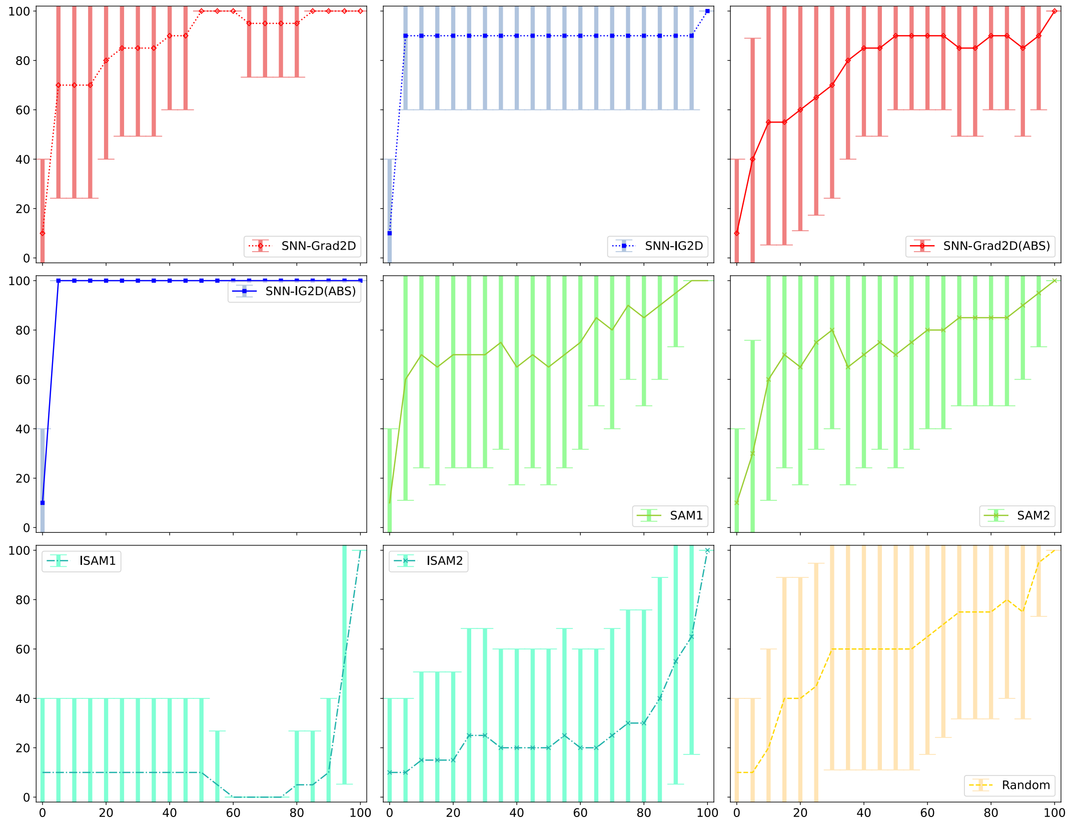

Figure S8. Error bars for Figure 4 (b) - % spikes inserted vs E[Winning Class]
